# Sex Differences and Caffeine Impact in Adenosine-Induced Hyperemia

**DOI:** 10.2967/jnumed.121.261970

**Published:** 2022-03

**Authors:** Martin Lyngby Lassen, Christina Byrne, Majid Sheykhzade, Mads Wissenberg, Preetee Kapisha Hurry, Anne Vibeke Schmedes, Andreas Kjaer, Philip Hasbak

**Affiliations:** 1 Department of Clinical Physiology, Nuclear Medicine and PET and Cluster for Molecular Imaging, Rigshospitalet and University of Copenhagen, Copenhagen, Denmark;; 2 Department of Drug Design and Pharmacology, Faculty of Health and Medical Sciences, University of Copenhagen, Copenhagen, Denmark;; 3 Department of Cardiology, Copenhagen University Hospital, Gentofte, Denmark; and; 4 Department of Biochemistry and Immunology, Lillebaelt Hospital, Vejle, Denmark

**Keywords:** caffeine, adenosine, myocardial flow reserve, PET, stress myocardial blood flow

## Abstract

Caffeine consumption before adenosine stress myocardial perfusion imaging (MPI) is known to affect the hemodynamic response and, thus, reduce the stress myocardial blood flow (MBF) and myocardial flow reserve (MFR) assessments. However, it is not clear if any sex-specific differences in the hemodynamic response after caffeine consumption exist. This study aimed to evaluate if such differences exist and, if so, their impact on MBF and MFR assessments. **Methods:** This study comprised 40 healthy volunteers (19 women). All volunteers underwent 4 serial rest/stress MPI sessions using ^82^Rb; 2 sessions were acquired without controlled caffeine consumption, and 2 sessions after oral ingestion of either 100 and 300 mg of caffeine or 200 and 400 mg of caffeine. For the caffeine imaging sessions, caffeine was ingested orally 1 h before the MPI scan. **Results:** Increase in plasma caffeine concentration (PCC) (mg/L) after consumption of caffeine was larger in women (MPI session without caffeine vs. MPI session with caffeine: women = 0.3 ± 0.2 vs. 5.4 ± 5.1, men = 0.1 ± 0.2 vs. 2.7 ± 2.6, both *P* < 0.001). Caffeine consumption led to reduced stress MBF and MFR assessments for men whereas no changes were reported for women (women [PCC < 1 mg/L vs. PCC ≥ 1 mg/L]: stress MBF = 3.3 ± 0.6 vs. 3.0 ± 0.8 mL/g/min, *P* = 0.07; MFR = 3.7 ± 0.6 vs. 3.5 ± 1.0, *P* = 0.35; men [PCC < 1 mg/L vs. PCC ≥ 1 mg/L]: stress MBF = 2.7 ± 0.7 vs. 2.1 ± 1.0 mL/g/min, *P* = 0.005; MFR = 3.8 ± 1.0 vs. 3.1 ± 1.4, *P* = 0.018). Significant differences in the stress MBF were observed for the 2 sexes (both *P* ≤ 0.001), whereas similar MFR was reported (both *P* ≥ 0.12). **Conclusion:** Associations between increases in PCC and reductions in stress MBF and MFR were observed for men, whereas women did not have the same hemodynamic response. Stress MBF was affected at lower PCCs in men than women.

## Visual Abstract

**Figure:**
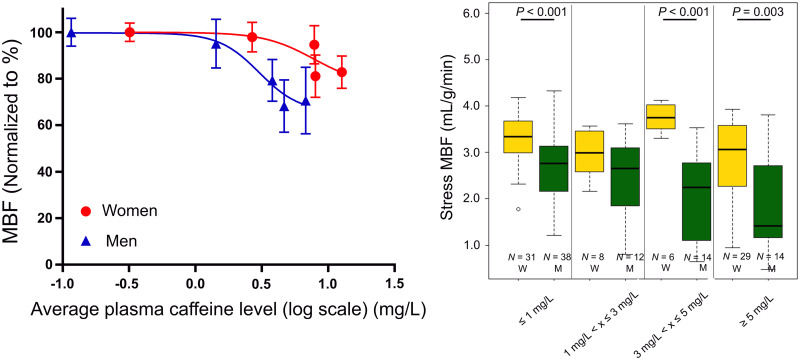

Myocardial blood flow (MBF) and myocardial flow reserve (MFR) assessments have become central in the clinical assessment of cardiac ^82^Rb in PET. The valid measures of stress MBF and MFR require a full hemodynamic response during the acquisition. Adenosine is a frequently used pharmacologic stressor that attaches to the A_2A_-adenosine receptor that mediates coronary vasodilation (*1*,*2*). When using adenosine as a pharmacologic stressor, patients are recommended to refrain from beverages, food, and analgesics containing caffeine for at least 12 h before the imaging session to avoid subpar stressing of the patients as caffeine nonselectively blocks the A_2A_-adenosine receptor (*1*–*3*). Consumption of caffeine has been shown to reduce the hemodynamic response even at plasma concentrations as low as 1 mg/L, which might introduce false-positive findings after the consequential reductions in the stress MBF and MFR (*4*–*6*). Although elevated caffeine plasma concentrations are known to affect the hemodynamic response when using adenosine as a stressing agent (*4*,*5*,*7*), it is unknown whether any sex-specific differences in the hemodynamic response exist (*5*,*8*).

This study aimed to evaluate the potential influence of sex on the association between plasma caffeine concentrations and stress MBF and MFR, respectively.

## MATERIALS AND METHODS

### Study Population

This study comprised 40 young healthy volunteers (19 women) (median age = 23 y, interquartile range [IQR] = 22; 25) recruited for rest/adenosine-stress myocardial perfusion ^82^Rb PET/CT from September 2016 to March 2017. Median volunteer weight was 70.0 kg (IQR = 62.0; 79.5 kg), with corresponding median body mass index of 22.0 (IQR = 20.5; 23.8). The volunteers underwent 4 serial PET/CT imaging sessions within 27 d (IQR = 17; 36), acquired with and without controlled caffeine consumption before the imaging session. Inclusion criteria were age >18 y, no participation in studies testing drugs, no regular consumption of medicine, no known medical condition, and no use of tobacco and euphoric substances (except alcohol) within 3 mo before study participation. Exclusion criteria were pregnancy, allergy, intolerance to theophylline or adenosine, any prior medical history of asthma, or inability to adhere to the study protocol. The Scientific Ethics Committee of the Capital Region of Denmark (protocol no. H-15009293) and the Danish Data Protection Agency approved this study, and all volunteers provided informed oral and written consent.

### Imaging Protocol

#### PET Acquisition

**FIGURE 1.**
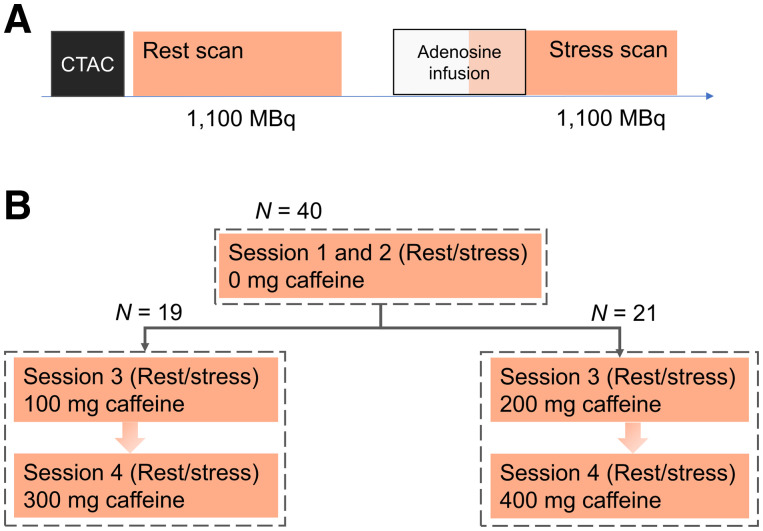
Imaging protocol. (A) Acquisition protocol for each of the 4 PET/CT imaging sessions. Both rest and stress scans were acquired over 6 min. (B) Study protocol for the 40 healthy volunteers. CTAC = computed tomographic attenuation correction.

The 40 healthy volunteers were divided into 2 groups, both undergoing 4 ^82^Rb PET/CT imaging sessions, each consisting of an ^82^Rb rest–stress protocol (Fig. 1A). All PET acquisitions, targeting injection doses of 1,100 MBq (30 mCi) ^82^Rb, were obtained in 3-dimensional mode on a 128-slice Biograph mCT PET/CT system (Siemens Healthineers) and stored in list-mode format (*9*). Pharmacologic stressing was obtained using adenosine infused at 140 mg/kg/min for 6 min with PET emission acquisition starting 2.5 min into the infusion (Fig. 1A). Before the rest scans, the volunteers underwent a low-dose CT for attenuation-correction purposes (120kVp; effective tube current, 26 mA [11 mAs quality reference]) acquired using a free-breathing protocol (*10*). The volunteers were instructed to abstain from caffeine at least 24 h before each of the 4 imaging sessions. All study volunteers underwent 2 imaging sessions without ingestion of caffeine and 2 imaging sessions after the ingestion of caffeine in a controlled setting using caffeine tablets (Fig. 1B). The caffeine tablets were dissolved in hot water and orally ingested 60 min before the rest perfusion scans. One-half of the volunteers had 2 serial imaging sessions with ingestion of 100 and 300 mg of caffeine, whereas the other half of the study volunteers underwent 2 serial imaging sessions after the ingestion of 200 and 400 mg of caffeine (Fig. 1B). The 4 imaging sessions were obtained in a randomized fashion. For this study, the plasma caffeine concentration is reported at the time of the stress myocardial perfusion imaging (MPI), thus, as an average of the measurements obtained at 75 and 90 min. All plasma caffeine concentrations were measured using high-performance liquid chromatography-mass spectrometry.

#### PET Reconstruction Protocol and Data Processing

The 6-min-long PET acquisitions were reconstructed into dynamic image series consisting of 18 frames (1 × 10 , 8 × 5, 3 × 10, 2 × 20, and 4 × 60 s) using the vendor iterative ordered-subset expectation-maximization 3-dimensional reconstruction method (2 iterations, 21 subsets), with corrections for time of flight and point-spread function. All data were smoothened using 6.5-mm gaussian postfiltering. MBF was calculated using the Lortie model (*11*), and MFR was calculated as the ratio of the stress and rest MBF (*12*) in dedicated software (QPET; Cedars-Sinai Medical Center (*13*)). We report MBF and MFR with and without corrections for the rate–pressure product (RPP), defined as RPP = (systolic blood pressure)/(heart rate); corrected rest MBF = (rest MBF)/RPP × 6,500 and corrected stress MBF = (stress MBF)/RPP ×8,600. Stress MBF and MFR above 3.0 were considered normal (*14*).

#### Repeatability

Test–retest repeatability of the rest and stress MBF and the MFR were calculated for the baseline scans (0 mg of caffeine ingested) with plasma caffeine concentrations < 1 mg/L using the coefficient of variance (*15*).

#### Coronary Vascular Resistance (CVR)

CVR was obtained for all scans using Equation 2 (*16*).

$$\mathrm{CVR}\;=\;0.33\;\times \frac{(2\;\times \;\text{diastolic}\;\text{blood}\;\text{pressure}\;+\;\text{systolic}\;\text{blood}\;\text{pressure})\;}{\text{MBF}}$$ (Eq. 1)

In Equation 2, MBF represents the scan-specific MBF. Both diastolic and systolic blood pressures were obtained during the PET emission acquisitions.

Regression plots for correlation between ingested caffeine and blood plasma concentration caffeine were calculated for all scans, using averaged plasma caffeine concentrations obtained at 75 and 90 min.

Concentration–response curves were obtained using a nonlinear curve-fit model, and the half-maximal relaxation (EC50, half-maximal effective concentration) was determined:

$$y\;=\;\text{bottom}\;+\;\frac{\left(\text{top}\;-\;\text{bottom}\right)}{1\;+\;10^{\left(\left(\mathrm{logEC}50\;-\;x\right)\;*\;\mathrm{slope}\right)}}$$ (Eq. 2)

### Statistical Analysis

Differences in the MBF and MFR were quantified using multivariable assessments (ANOVA) in R (GNU project). Descriptive analyses of continuous values were reported as mean ± SD and range or median and IQR. Two-tailed *P* values of less than 0.05 were considered statistically significant. All data were checked for normality using the Sharpio–Wilk test. Dependency of MFR and caffeine ingestion was determined using a 2-way ANOVA, with *P* values less than 0.05 being considered significant. Regressional analyses between plasma caffeine concentration and ingested caffeine were obtained for both sexes.

## RESULTS

### Study Population

A total of 40 volunteers underwent 4 serial PET/CT scans within 27 d (IQR = 17; 36). The volunteers had a median age of 23 y (IQR = =22; 25), and 19 (47.5%) were women. Average body mass index of the volunteers was 22.7 ± 3.2 (women = 21.9 ± 3.1, men = 23.5 ± 3.0, *P* = 0.08). The median habitual daily coffee consumption was 2 cups (IQR = 1.5; 3) (women = 2, IQR = 1.5; 3 and men = 2, IQR = 1.5; 3, *P* = 0.59) per day, representing a median caffeine intake from coffee of 200 mg/d (IQR = 150–300).

Of the 40 volunteers, 1 volunteer (man) was excluded due to failure to comply with the protocol. In addition, 4 scans were excluded because of significant motion during the scans (0-mg scans: *n* = 2; 200-mg scan: *n* = 1, 400-mg scan: *n* = 1). In total, 152 of the 160 acquired imaging sessions (95%) were used for the subsequent analyses.

### Rest MBF

**FIGURE 2.**
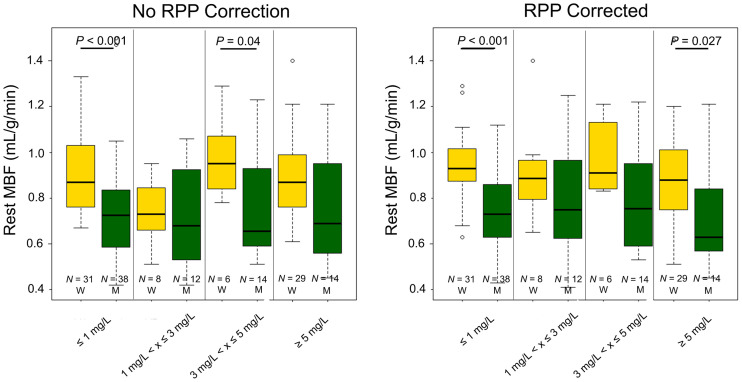
Rest MBF obtained in the volunteers sorted by sex and plasma caffeine concentration with and without RPP correction. W = women; M = men; RPP = rate pressure product; N = number of MPI sessions fulfilling the criteria.

Rest MBF assessments are shown in Figure 2. Significant differences in the rest MBF assessments for the 2 sexes were observed for MPI sessions with plasma caffeine concentrations ≤ 1 mg/L (*P* < 0.001) (no caffeine ingested) and ≥ 5 mg/L (*P* = 0.027) (caffeine ingested). Reduced heart rates were observed in the women after ingestion of caffeine whereas men had stable heart rates (Table 1). Similar test–retest repeatability coefficients were reported for the men and women (16.1% and 13.5% [*P* = 0.42], respectively) (Table 2). Women had higher diastolic pressure than men (62.3 ± 6.4 vs. 59.7 ± 7.8 mm Hg, *P* = 0.02) whereas lower systolic blood pressure was reported for women (104.6 ± 12.1 vs. 112.3 ± 12.4 mm Hg, *P* < 0.001) **(**Tables 3 and 4**)**. No difference in RPP was observed between the 2 sexes (RPP rest: 6,394 ± 1,400 vs. 6,481 ± 1,488 [*P* = 0.72]) (Table 5). At rest, men had increased CVR when compared with women (Table 6).

**TABLE 1.** Heart Rate (Beats per Minute) Obtained During Scans Using 4 Levels of Plasma Caffeine Concentration

	Plasma caffeine concentration (mg/L)			
PCC	≤1	1 < x ≤ 3	3 < x ≤ 5	>5
Rest				
Women	62.4 ± 10.8 (31)	51.4 ± 7.3* (31)	68.0 ± 14.3 (6)	60.9 ± 10.6 (29)
Men	60.2 ± 13.0 (38)	53.8 ± 12.1 (12)	55.7 ± 9.0^†^ (14)	60.1 ± 9.3 (14)
Stress				
Women	92.5 ± 14.4	79.3 ± 23.6*	91.5 ± 9.7	78.2 ± 15.2*
Men	80.9 ± 19.7^†^	73.3 ± 18.8	74.4 ± 15.8^†^	70.6 ± 13.2

* Significant variations in the heart rate between the baseline scans (plasma caffeine concentration < 1 mg/L) and studies with increased plasma caffeine concentrations.

^†^Intersex differences in the heart rate observed for the respective plasma caffeine concentrations.

Numbers given in parentheses for the rest scans indicate the number of MPI sessions fulfilling the criteria. Differences were considered significant for *P* < 0.05.

PCC = plasma caffeine concentration.

**TABLE 2.** Test–Retest Repeatability (Measured as Coefficient of Variation) for Baseline MPI Sessions (0 mg of Caffeine Ingested)

Coefficient of variation	Rest	Stress	MFR
Women (*n* = 19)	13.5%	10.6%	12.9%
Men (*n* = 20)	16.1%	18.4%	20.6%
Combined (*n* = 39)	15.8%	15.3%	17.8%

No differences in test–retest repeatability was observed between the 2 sexes.

**TABLE 3.** Diastolic Blood Pressure Obtained During Scans Using 4 Levels of Plasma Caffeine Concentration

	Plasma caffeine concentration (mg/L)			
PCC	≤1	1 < x ≤ 3	3 < x ≤ 5	>5
Rest				
Women	60.9 ± 6.5 (31)	62.3 ± 4.6 (8)	60.5 ± 8.3 (6)	64.2 ± 6.4 (29)
Men	57.9 ± 9.1 (38)	61.2 ± 7.3 (12)	60.4 ± 5.7 (14)	62.2 ± 5.7 (14)
Stress				
Women	60.8 ± 9.0	68.4 ± 10.2	60.4 ± 5.7	66.3 ± 8.8*
Men	54.7 ± 9.0^†^	61.0 ± 9.8*	58.7 ± 8.1	59.5 ± 6.8^†^

* Significant variations in the diastolic blood pressure between the baseline scans (plasma caffeine concentration < 1 mg/L) and studies with increased plasma caffeine concentrations.

^†^Intersex differences in the diastolic blood pressure observed for the respective plasma caffeine concentrations.

Numbers given in parentheses for the rest scans indicate the number of MPI sessions fulfilling the criteria. Differences were considered significant for *P* < 0.05.

PCC = plasma caffeine concentration.

**TABLE 4.** Systolic Blood Pressure Obtained During Scans Using 4 Plasma Caffeine Concentration

	Plasma caffeine concentration (mg/L)			
PCC	≤1	1 < x ≤ 3	3 < x ≤ 5	>5
Rest				
Women	102.6 ± 9.9 (31)	103.9 ± 8.1 (8)	99.2 ± 17.3 (6)	108.7 ± 13.6 (29)
Men	108.9 ± 10.7 (38)	112.6 ± 10.0 (12)	112.5 ± 10.7 (14)	114.9 ± 9.3 (14)
Stress				
Women	92.5 ± 14.4	96.5 ± 17.7	102.3 ± 6.4	105.9 ± 12.3
Men	107.0 ± 12.7^†^	110.6 ± 13.5	112.1 ± 10.1^†^	111.5 ± 12.1

* Within-sex variations in the systolic blood pressure between the baseline scans (plasma caffeine concentration < 1 mg/L) and studies with increased plasma caffeine concentrations.

^†^Intersex differences in the systolic blood pressure observed for the respective plasma caffeine concentrations.

Numbers given in parentheses for the rest scans indicate the number of MPI sessions fulfilling the criteria. Differences were considered significant for *P* < 0.05.

PCC = plasma caffeine concentration.

**TABLE 5.** RPP Stratified for the 2 Sexes at 4 Levels of Plasma Caffeine Concentration

	Plasma caffeine concentration (mg/L)			
PCC	≤1	1 < x ≤ 3	3 < x ≤ 5	>5
Rest				
Women	6,422 ± 1,381 (31)	5,329 ± 797* (8)	6,626 ± 1,253 (6)	6,627 ± 1,475 (29)
Men	6,504 ± 1,381 (38)	6,100 ± 1,739 (12)	6,429 ± 1,075 (14)	6,786 ± 1,447 (14)
Stress				
Women	9,624 ± 2,013	7,480 ± 2,093*	9,328 ± 702	8,333 ± 2,105*
Men	8,742 ± 2,350	8,208 ± 2,757	8,279 ± 1,666	7,770 ± 1,703

* Sex-specific differences between the baseline scan (0 mg) and the respective plasma caffeine concentrations.

Numbers given in parentheses for the rest scans indicate the number of MPI sessions fulfilling the criteria. Differences were considered significant for *P* < 0.05.

PCC = plasma caffeine concentration.

**TABLE 6.** CVR Obtained for Rest and Stress Using 4 Levels of Plasma Caffeine Concentration

	Plasma caffeine concentration (mg/L)			
PCC	≤1	1 < x ≤ 3	3 < x ≤ 5	>5
Rest				
Women	82.9 ± 19.4 (31)	85.7 ± 16.8 (8)	77.0 ± 18.5 (6)	92.6 ± 21.1 (29)
Men	105.5 ± 30.7* (38)	101.3 ± 26.5 (12)	102.1 ± 27.6 (14)	152.9 ± 25.9*^†^ (14)
Stress				
Women	25.8 ± 5.9	23.0 ± 7.2	21.8 ± 1.4	23.6 ± 8.8
Men	27.5 ± 8.2	33.0 ± 15.2	45.2 ± 25.6*^†^	51.8 ± 14.8*^†^

* Significant differences for men and women.

^†^Sex-specific differences between the baseline scan (0 mg) and the respective plasma caffeine concentrations.

Numbers given in parentheses for the rest scans indicate the number of MPI sessions fulfilling the criteria. Differences were considered significant for *P* < 0.05.

PCC = plasma caffeine concentration.

### Stress MBF

Women had higher heart rate and diastolic blood pressure, whereas systolic blood pressure was decreased when compared with men (heart rate: 85.1 ± 16.8 vs. 76.2 ± 18.0 beats per minute, diastolic blood pressure: 63.9 ± 9.1 vs. 57.3 ± 8.8 mm Hg, systolic blood pressure: 103.6 ± 12.1 vs. 109.4 ± 12.3 mm Hg, all *P* < 0.005) (Tables 1, 3, and 4). No difference was observed for RPP in women and men (8,833 ± 2,098 vs. 8,318 ± 2,155, *P* = 0.14, Table 5).

**FIGURE 3.**
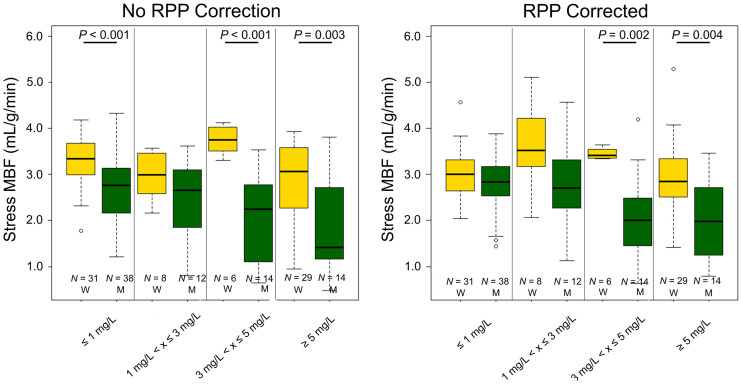
Stress MBF obtained in the volunteers sorted by sex and plasma caffeine concentration, with and without RPP correction. W = women; M = men; RPP = rate pressure product; N = number of MPI sessions fulfilling the criteria.

**FIGURE 4.**
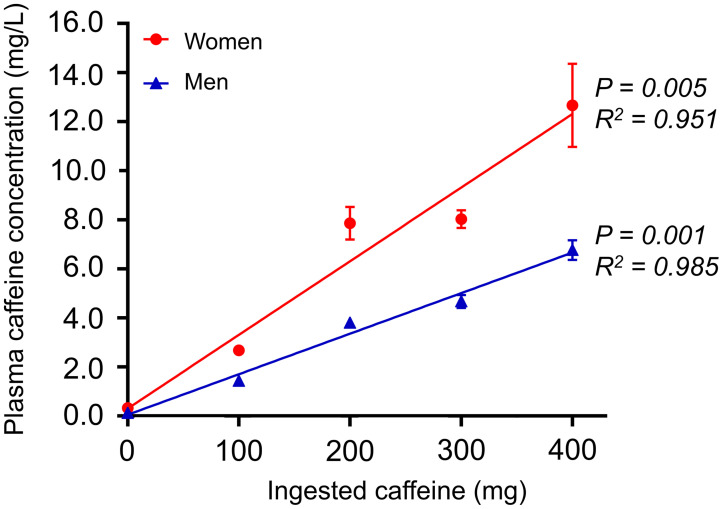
Relationship between ingested caffeine (mg) and the corresponding plasma caffeine concentrations (mg/L). The plasma caffeine concentrations obtained at 75 and 90 min are given as mean ± SEM in both men (closed blue triangles, *n* = 9–20) and women (closed red circles, *n* = 9–18).

**FIGURE 5.**
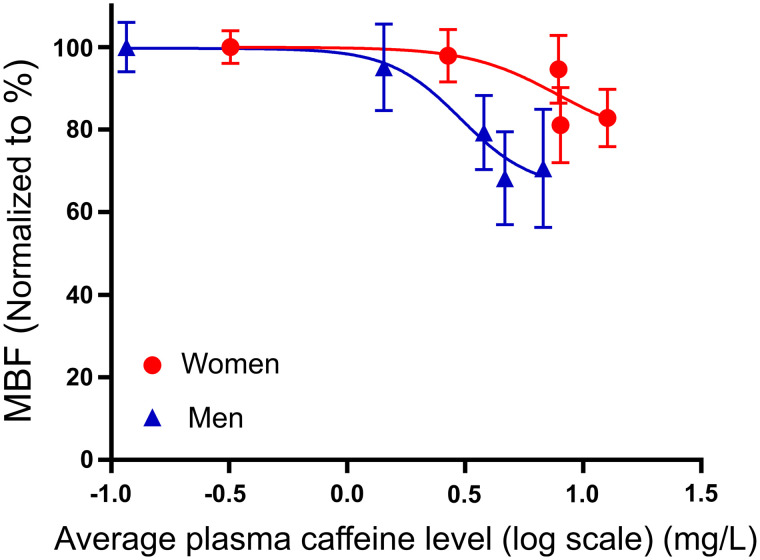
Relationship between average plasma concentration of caffeine [mg/L] (log) and stress MBF. Points represent mean values, and vertical lines indicate SEM. MBF values were normalized to the maximum stress MBF obtained for the individual volunteers (men, closed blue triangles, *n* = 9–20, and women, closed red circles, *n* = 9–18, respectively).

Increased stress MBF assessments were reported for women when compared with men (stress MBF [mL/g/min]: women vs. men [plasma caffeine concentration < 1 mg/L, no caffeine ingestion]: 3.3 ± 0.6 vs. 2.7 ± 0.7, [plasma caffeine concentration ≥ 1 mg/L, caffeine ingestion]: 3.0 ± 0.8 vs. 2.1 ± 1.0, both *P* < 0.001) (Fig. 3). Similar test–retest repeatability coefficients were reported for the men and women (18.4% and 10.9% (*P* = 0.29), respectively) (Table 2). Significant changes in the hemodynamic response were observed for the men. In contrast, consistent stress MBF assessments were observed for women at plasma caffeine concentrations below 5 mg/L (Fig. 3). Corresponding to the change in the hemodynamic response, CVR was observed to increase for men even at low plasma caffeine concentrations, whereas no changes in CVR were observed for women (Table 6). Linear correlations between ingested caffeine and plasma concentrations of caffeine in both men and women were observed (Fig. 4). Increased plasma caffeine concentrations were measured in women compared with men for the same doses of ingested caffeine. Furthermore, an almost 3-fold increase in the sensitivity to caffeine was observed in men, with consequential reductions in the stress MBF assessments (EC50 [mg/L]: men ≈ 3, women ≈ 8) (Fig. 5). Multivariable analyses including stress MBF, sex, and plasma caffeine concentrations revealed that both caffeine plasma concentration (*P* < 0.001), sex (*P* < 0.001), and the interaction of the 2 (*P* =0.049) had a significant impact on stress MBF assessments.

### MFR

**FIGURE 6.**
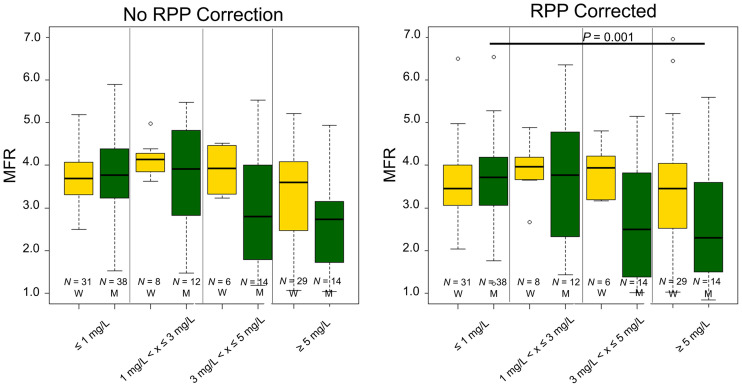
MFR obtained in the volunteers sorted by sex and plasma caffeine levels. W = women; M = men; RPP = rate pressure product; N = number of MPI sessions fulfilling the criteria.

Comparable MFR assessments were observed for women and men (Fig. 6), with similar repeatability coefficients for the 2 sexes (women = 12.9%, men = 20.6% [*P* = 0.21]) (Table 2). However, MFR was reduced in men when plasma caffeine concentrations exceeded 5 mg/L, whereas women had stable MFR at all plasma caffeine concentrations (Fig. 6). On an individual basis, MFR might be reduced even at plasma concentrations as low as 1.2 mg/L in men and 7.1 mg/L in women. Multivariable analyses including MFR, sex, and plasma caffeine concentrations revealed that sex and caffeine plasma concentration (both *P* < 0.001), as well as the interaction of sex and plasma caffeine concentration (*P* = 0.049), had a significant impact on the MFR assessments.

## DISCUSSION

In this randomized controlled crossover trial, we evaluated the impact of plasma caffeine concentrations on stress MBF and MFR assessments in healthy volunteers. The main finding was a sex-specific response in stress MBF and MFR assessments when the volunteers had stress perfusion imaging after caffeine consumption. Multivariable analyses of the data revealed that sex, caffeine concentration, and the interaction of the 2 were strongly associated with changes in stress MBF and MFR. Hence, in healthy volunteers, caffeine intake was associated with a different hemodynamic response in men and women with consequential differences in the stress MBF and MFR assessments. Men were found more sensitive to caffeine concentration, where plasma caffeine concentrations as low as 1.2 mg/L might affect the perfusion estimates compared with 7.1 mg/L in women.

Baseline rest and stress MBF (plasma caffeine concentrations < 1 mg/L) were increased in women, which might be explained by the estrogenic effect on the vascular tone (*17*). Several studies have shown an evident trend for higher plasma caffeine and lower plasma paraxanthine (the most active caffeine metabolite) concentrations in women as compared with men, suggesting that women metabolize caffeine slower than men. Women with higher estrogen levels have been reported to have decreased cytochrome P450 1A2 enzyme activity and decreased caffeine clearance (*18*). Furthermore, a study has reported that women have 25% higher adenosine A_1A_ (at messenger RNA level) receptor and 40% lower A_2A_ receptor expression (at messenger RNA level) than men and ovariectomized women (*19*). Thus, one can speculate that the differences in antagonistic potency of caffeine observed between men and women in our study is partially attributed to sex-related expression, adenosine receptor reserve, and intracellular signaling. However, the mechanism(s) involved are likely to be more complex and need to be scrutinized in future studies.

Stress MBF and MFR were observed to fluctuate for the women at different plasma caffeine concentrations. These effects may be caused by the low number of measurements obtained for the plasma caffeine concentrations ranging between 1 and 5 mg/L and, thus, statistical noise. The general reduction in stress MBF for scans obtained after caffeine consumption reported for the male volunteers was in concurrence with previous studies (*5*,*7*,*16*). Test–retest repeatability was tested for the baseline scans for all subjects with concurrent plasma caffeine concentrations < 1 mg/L. In this context, test–retest repeatability was found in concordance with previous studies evaluating the short-term variation in rest MBF (*15*). The concurrent test–retest repeatability measures, therefore, suggest that reductions in the MBF and MFR assessments observed for high plasma caffeine concentrations were introduced by the ingestion of caffeine. This finding is supported by the observed physiologic responses to caffeine ingestion. In concordance with previous studies, women had greater increases in diastolic blood pressure after caffeine administration than men (Table 3) (*20*,*21*). One explanation for this effect may be men have a more sensitive baroreflex than women (*20*,*21*). Further, sex differences in hemodynamic response to caffeine may be related to sex steroid hormone concentrations (*22*–*24*). When taken together, these studies suggest that men are more responsive to caffeine than women, a hypothesis supported by the findings in this study since caffeine had a greater potency in reducing stress MBF in men than women (Fig. 5). Caffeine competitively blocks all adenosine receptors (A_1_, A_2A_, A_2B_, A_3_), resulting in a compensatory increase in adenosine concentration, which in turn stimulates circulating chemoreceptors and other receptors (*25*). In adult mammals, it has been suggested that A_2A_ receptors are implicated in O_2_ sensing by carotid glomus cells (chemoreceptors) and are involved in the transduction mechanisms of O_2_ sensing in carotid bodies. Therefore, excessive adenosine can activate chemoreceptors via binding to A_2A_ receptors located in carotid bodies, thereby increasing sympathetic outflow of catecholamines leading to an increase in vascular resistance, and renin secretion (*26*). In this study, CVR was found to increase in men when the plasma caffeine concentration increased, whereas women had no significant changes in the CVR after ingestion of caffeine (Table 6). Sex differences in the clearance of plasma-protein-binding and differences in the volume of distribution may also explain some of the differences in the perfusion response to caffeine between sexes (*27*). Regional conditions favor regadenoson and dipyridamole over adenosine as the drugs of choice. Although only adenosine was evaluated in this study, it is likely that our findings can also apply to regadenoson and dipyridamole.

### Study Limitations

This study was conducted in healthy young volunteers with normal cardiac perfusion without perfusion defects, and we cannot rule out that results would have differed in the typical patient population undergoing cardiac ^82^Rb PET testing. Patient populations are often elderly and have an intermediate likelihood of ischemic heart disease in addition to lower sex hormone concentrations, caffeine pharmacokinetics, and pharmacodynamics. Combined, these differences might cause different hemodynamic responses than those reported in this study. However, we find that an establishment of sex-specific data in young normal volunteers is important before investigating the impact of caffeine consumption in an elderly cohort in whom comorbidities and coronary artery disease are common. Another limitation is that our study was conducted in a single center using only 1 imaging system, which might affect the overall MBF and MFR assessments.

## CONCLUSION

In healthy volunteers, we found that caffeine consumption before MPI affected men and women differently. Associations between plasma caffeine and significant reductions in stress MBF and MFR were found at lower plasma caffeine concentrations in men than in women.

## DISCLOSURE

No potential conflict of interest relevant to this article was reported.

## Notes

KEY POINTS **QUESTION:** Does caffeine before stress MPI affect the hemodynamic response differently in women and men? **PERTINENT FINDINGS:** Caffeine consumption affects the hemodynamic response at a lower plasma caffeine concentration in men than women, with the stress MBF being affected with plasma concentrations of 1.2 mg/L in men as compared with 7.4 mg/L in women. The dose–response of caffeine plasma concentration and obtained stress MBF assessments revealed significantly increased sensitivity to caffeine in men when compared with women. **IMPLICATIONS FOR PATIENT CARE:** Caffeine consumption less than 12 h before MPI sessions might lower the stress MBF assessments. At comparable plasma caffeine concentrations, men seem to be particularly affected and should be encouraged to abstain from caffeine for longer times than women before MPI.
